# Revealing spatio-spectral electroencephalographic dynamics of musical mode and tempo perception by independent component analysis

**DOI:** 10.1186/1743-0003-11-18

**Published:** 2014-02-28

**Authors:** Yuan-Pin Lin, Jeng-Ren Duann, Wenfeng Feng, Jyh-Horng Chen, Tzyy-Ping Jung

**Affiliations:** 1 Institute for Neural Computation and Institute of Engineering in Medicine, University of California, San Diego, La Jolla, CA, USA; 2 Biomedical Engineering Research and Development Center, China Medical University Hospital, Taichung, Taiwan; 3 Department of Neurosciences, University of California, San Diego, La Jolla, CA, USA; 4 Department of Psychology, SooChow University, Suzhou, Jiangsu, China; 5 Department of Electrical Engineering, National Taiwan University, Taipei, Taiwan

**Keywords:** EEG, Independent component analysis, Musical structure

## Abstract

**Background:**

Music conveys emotion by manipulating musical structures, particularly musical mode- and tempo-impact. The neural correlates of musical mode and tempo perception revealed by electroencephalography (EEG) have not been adequately addressed in the literature.

**Method:**

This study used independent component analysis (ICA) to systematically assess spatio-spectral EEG dynamics associated with the changes of musical mode and tempo.

**Results:**

Empirical results showed that music with major mode augmented delta-band activity over the right sensorimotor cortex, suppressed theta activity over the superior parietal cortex, and moderately suppressed beta activity over the medial frontal cortex, compared to minor-mode music, whereas fast-tempo music engaged significant alpha suppression over the right sensorimotor cortex.

**Conclusion:**

The resultant EEG brain sources were comparable with previous studies obtained by other neuroimaging modalities, such as functional magnetic resonance imaging (fMRI) and positron emission tomography (PET). In conjunction with advanced dry and mobile EEG technology, the EEG results might facilitate the translation from laboratory-oriented research to real-life applications for music therapy, training and entertainment in naturalistic environments.

## Background

Understanding the underlying neural mechanisms associated with music perception is an intriguing but challenging task. Several studies have demonstrated that music can induce or enhance emotional responses [1-3]. However, the details regarding neural correlates of music perception are still largely unclear as music perception is a complex cognitive task involving the perception and integration of various structural components of music, such as melody, harmony, pitch, rhythm, tempo, mode, and timbre [4-7]. Conveying emotion in music by manipulating and integrating these musical structures is intuitively plausible [4,8].

Most music perception studies in the literature have exploited functional neuroimaging modalities such as functional magnetic resonance imaging (fMRI) and positron emission tomography (PET) to assess cerebral correlates of emotional responses to musical structures, such as mode (the specific subset of pitches) and tempo (the number of beats per minute), which are believed to be the major musical structures affecting the perception of emotional valence [5,9,10]. An easily imaginable example is the distinction between happy and sad music, as happy music is typically associated with fast tempo and major mode, whereas sad music is associated with slow tempo and minor mode [5,10,11]. Several studies have explored the neural basis of musical mode perception to elucidate its correlation with music-induced emotion. For instance, a PET study [1] exposed subjects to varying harmonic chord structures to make music sound more or less consonant or dissonant in order to evaluate how regional cerebral blood flow (rCBF) changes in distinct paralimbic and neocortex regions as a function of dissonance and/or perceived pleasantness/unpleasantness. Another fMRI study [7] reported that blood-oxygen-level dependent (BOLD) signals increased in the parietal and occipital regions during harmonic melody that was strongly associated with emotional affect and intensity. Green *et al.*[11] reported that minor-mode melodies increased activity in the limbic structures. Another fMRI study [10] demonstrated that changes in musical mode and tempo involved the orbitofrontal and cingulate cortices, which are known to intervene in emotion processing. A magnetoencephalographic (MEG) study [12] demonstrated that the activity in the motor-related structures correlated with measures of musical rhythmicity.

Comparing the studies using high-cost neuroimaging techniques, assessing the musical structure-related brain dynamics through electroencephalography (EEG) has gained increasing attention in the past few years. For example, Tsang *et al.*[13] showed that music composed in major mode and played at a fast tempo primarily activated the left frontal regions, which were known to be related to positive-valence emotion, whereas music played at a slow tempo and minor mode activated the right frontal regions, which were associated with negative-valence emotion. Tian *et al*. [14] very recently reported the frontal midline theta decreased with increased arousal level of musical tempo. The frontal midline theta has been reported to activate during musical emotion perception [15,16]. Several event-related potential (ERP) studies directly correlated the temporal activations in the perception of musical mode and rhythm [17-19]. To avoid volume conduction in EEG recording, Cong *et al*. [20] applied independent component analysis (ICA) to isolate the activations of the brain sources associated with musical tonal and rhythmic waveforms. They reported two brain regions whose theta and alpha activities sparsely and distinctly associated with the musical attributes.

Compared to other neuroimaging modalities (such as fMRI, PET, and MEG), EEG modality is lightweight, portable, easy-of-use, and low-cost. Furthermore, EEG is the only modality that does not require the head and body to be fixed during measurement. Recent advances in dry and wireless EEG systems [21,22] further promoted the translation from laboratory-oriented neuroscience research to clinical and entertainment applications in real-life environments. A laboratory-level EEG headset usually requires lengthy skin preparation by skilled technicians and applications of conductive (wet) gels, tangled cables, a head box and a data logger. All these settings very likely lead to movement-constrained behaviors during EEG recording. This bulky and tethered EEG headset is thus impractical for EEG applications in real-world environments. In contrast, a dry and mobile EEG system featuring dry electrodes and wireless telemetry has bridged the gap between well-controlled laboratory and ecologically valid environments by allowing users to quickly and easily wear the EEG headset for recording and monitoring. Considering the above issues, EEG modality is the clear modality of choice for translating the music-related EEG results to practical real-life applications.

Studying EEG dynamics typically relies on the calculation of temporal and/or spectral dynamics from signals recorded directly from the scalp. Due to volume conduction, EEG data recorded at the scalp are linear mixtures of electrical potentials projected from multiple distinct cortical domains and non-brain artifacts arising from eye blinking, lateral eye movement, muscle tension, etc. [23]. The signal-mixing process makes it difficult to link recorded EEG signals with specific brain functions [24]. ICA, which is used to estimate statistically independent sources from the mixtures, is effective not only for isolating non-cortical activity, but also for separating temporally independent and spatially fixed sources [25,26]. Several studies [27-29] have demonstrated the effectiveness of ICA on improving signal-to-noise ratio (SNR) of activities of interest. Jung *et al*. [29] reported that applying ICA to the analysis of sets of single trials from event-related EEG experiments can increase the information available from ERP data. Lemm *et al*. [28] also demonstrated that ICA improved the SNR of single-trial somatosensory evoked potentials from multichannel EEG-recordings. Recently, Wang *et al*. [27] reported that ICA was capable of separating motor-related mu rhythm from the background alpha activity, which in turn enhanced the SNR of motor-imagery induced brain rhythm in comparison with channel-level EEG signals. The ICA has been widely applied to multi-channel EEG signals when exploring brain dynamics in human cognition, including motion sickness [30], emotion imagery [31], musical emotion [15], musical perception [20] and visual perception [29,32]. The resultant source contributions would tend to have focal and distinct brain activity compatible with physiological responses [23]. Notably, although a recent study [20] also proposed the use of ICA for assessing musical structures, it only addressed the theta and alpha activities and did not correlate them with musical tempo. In contrast, this study fully explored the EEG dynamics in five frequency bands across different brain processes that are associated with music and emotion perception [18,33-36].

This study aimed to employ ICA to decompose multi-channel scalp EEG data into spatially independent brain sources that are associated with the perception of musical mode and tempo during music listening. This study addressed three specific issues: 1) what independent brain processes are associated with musical mode or tempo perception, 2) whether these brain processes are consistent with previously reported music-related evidences obtained by other neuroimaging modalities, and 3) how these spatio-spectral dynamics of different brain networks are modulated by the musical mode and tempo. The resultant music-modulated EEG dynamics of the present study would provide foundational insights into the relationship between the brain's electrical activity and musical structures.

## Methods

### Subjects

Twenty-four healthy right handed volunteers (fourteen males, ten females; age 24.61 ± 2.52 yr.) participated in this study. Most subjects were undergraduate or graduate students in the College of Electrical Engineering and Computer Science or in the College of Engineering at National Taiwan University. They had minimal formal musical training and could thus all be considered as non-musician. All subjects gave written consent before participated in the study, which was approved by the Human Research Protections Program of National Taiwan University, Taipei, Taiwan.

### Experimental procedure

During the experiment, the subjects listened to sixteen 30-s excerpts from Oscar-winning film soundtracks [37]. Sixteen music excerpts were randomly selected without replacement for use in a four-run experiment; in each run, four music excerpts were played interleaved with 15-sec silent rests. Thus, all subjects would be listening to all sixteen music excerpts, but in a random sequence, during their EEG sessions. Subjects were instructed to keep their eyes closed, to minimize their head/body movements, and to remain seated throughout the entire music-listening experiments. Since the music experiment did not instruct subjects to gaze at a fixation cross presented on a screen and to simultaneously rate the music during the recording, closing eyes helped them to attentively yet comfortably listen to music in the hour-long experiment. Notably, the subjects were not instructed to identify specific musical structures. Each experiment thus obtained sixteen 30-s EEG segments for correlation analysis with musical structures.

### Musical structure extraction

Unlike the subjective rating of musical structures by musical experts used in a study [4], this study objectively characterized the mode (major or minor) and tempo (fast or slow) of the music excerpts using MIRToolbox [38], a MATLAB toolbox quantitatively analyzes musical structures of interest directly from an audio file. The MIRToolbox has been used for musical feature extraction in several music studies [39,40]. The MIRToolbox rated the musical mode on a scale from -1 to 1, in which positive values indicate major mode and negative values indicate minor mode. The estimated tempo of music excerpts used in this study ranged from 90 to 168 bpm. Based on the estimates obtained by MIRToolbox, the sixteen music excerpts used in this study were categorized as fast (>125 bpm, 8/16) or slow (8/16) in tempo and as major (10/16) or minor (6/16) in mode.

### EEG data acquisition and preprocessing

A 32-channel EEG system (Neuroscan, Compumedics Ltd., Australia) was used to record the EEG and electrooculogram (EOG) signals. The 30 scalp electrodes were placed according to the modified International 10-20 system and referred to the linked mastoids (average of A1 and A2). The EEG and EOG signals were sampled at 500 Hz with a band-pass filter (1–100 Hz) and a 60 Hz notch filter to avoid power-line contamination. The impedances of all electrodes were kept below 10 KOhm. Since subjects were instructed to keep eyes closed and remain still throughout the experiment, only a small portion of data with rare but large motion artifacts presented during the data recording. This study manually removed such transient artifacts (on average, only 0.56 ± 0.86% of the sample points across 24 subjects were removed from further analysis).

### Independent component analysis and clustering

The ICA was applied to decompose multi-channel EEG data into maximally statistically independent components (ICs), which were obtained by using the extended infomax ICA algorithm implemented in the EEGLAB toolbox [41]. In this study, ICA was only applied to 16 30-s music-listening EEG segments for each subject, that is, the 15-sec signals of rest periods between music excerpts were not included in the decomposition. The ICA finds an ‘unmixing’ matrix W that linearly unmixes the multi-channel EEG data X into a temporally independent source matrix U, where U = WX. The rows of estimated source matrix U (“component activations”), are the time courses of the corresponding ICs, its columns indicate the time points. This study decomposed 30 ICs from 30 channels of the EEG signals (2 EOG channels excluded) for each subject, where the ICA unmixing matrix W was trained with a stopping criterion of total weight change of 10^-7^. The columns of the inverse unmixing matrix, W^-1^, represented the relative projection strengths of the ICs onto each scalp sensor. Relative projection strengths were interpolated and color-coded to form a scalp map (topography) associated with each component [26]. To localize the sources of independent components, DIPFIT2 routine, a plug-in in EEGLAB, was used to fit single-dipole models to the IC scalp topographies by using a standardized boundary element head model (BEM) [42]. If the residual variance of the single-dipole fit to the scalp projection of an IC exceeded 15%, the IC was removed from further analysis [23]. ICs with an equivalent dipole located outside the model brain volume were also excluded. Dipole locations were mapped to a 3D brain image by co-registering them with the Montreal Neurological Institute (MNI) brain template.

Lastly, the consistency of ICs from multiple subjects was categorized using *k*-means clustering method and visual inspection to semi-automatically group similar components across subjects into distinct IC clusters. *K*-means aims to partition resultant ICs into *k* clusters by minimizing the average squared Euclidean distance of observations from their cluster centers. The Euclidean distance was calculated from a combination of scalp maps, dipole locations of ICs, and power spectra of the component activations. These features formed a 38-dimensional feature vector (30 for scalp map, 5 for spectral band power, and 3 for 3-D dipole location). As reported in [23], applying ICA decomposition to 31-channel data usually yields 5 to 15 physiologically plausible components of which the dipole models can account for more than 85% of the variance of component activation maps. The predetermined number of clusters was thus initially set to 15 in the current study. After the automatic clustering finished, we visually inspected and verified the consistency of the ICs within each cluster in terms of scalp maps, spectral profiles, and equivalent dipole locations. Among the grouped clusters, several stereotyped ICs accounting for eye blinks, lateral eye movements, and sporadic muscle tension [23,43] were discarded. The components with homogeneous scalp maps and within-brain dipole locations which are commonly considered as neurophysiological interpretable brain sources were included for further analysis.

### Analysis of the associations between musical structures and EEG

Short-time Fourier transform (STFT) with a 500-point non-overlapping Hamming window was applied to the time course of each component to estimate the component spectrogram. The spectra were then grouped by averaging the power spectra within characteristic EEG frequency bands, namely delta (1-3 Hz), theta (4-7 Hz), alpha (8-13 Hz), beta (14-30 Hz) and gamma (31-50 Hz). For each frequency band, the mean baseline power was derived from the first five seconds of music presentation. The logarithmic spectral time series data for each IC were normalized by subtracting the baseline power and dividing by the standard deviation from the spectra. We then averaged the normalized spectral time series across grouped ICs for each 30-s music trial. This procedure returned a data length of 16 (the number of music trials), representing each of five frequency bands of the IC cluster of interest, for further statistical analysis. To investigate the musical mode effects on EEG spatio-spectral activities, the analysis of covariance (ANCOVA) was performed (with *p* < 0.05) separately for each spectral band (delta, theta, alpha, beta, and gamma) of each IC cluster on the factor of mode (major versus minor) given another factor, *i.e.*, tempo, as a covariate. The procedures of ANCOVA repeated to investigate the musical tempo effects on EEG activities while using tempo as factor and treating mode as a covariate.

## Results

### Independent component clusters and component spectra

**Figure 1:**
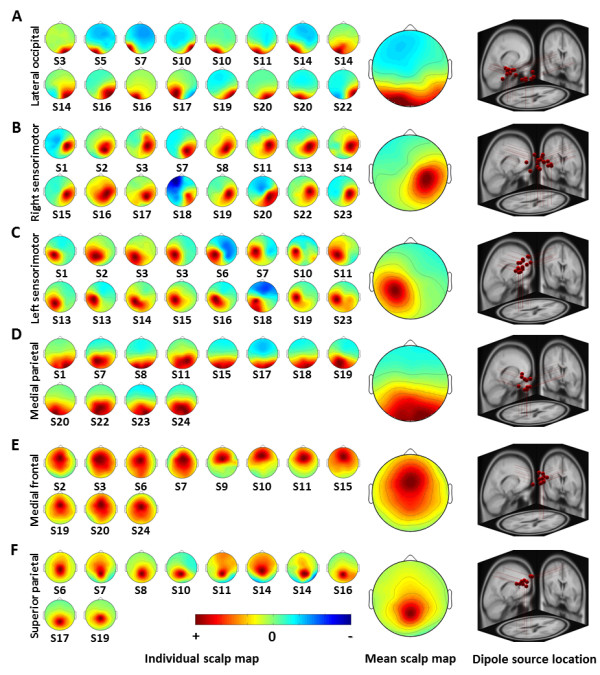
**Scalp maps and dipole source locations of six independent component clusters across twenty-four subjects.** Left panel: Individual scalp maps indicate components found by ICA for a single subject. Middle panel: Mean scalp maps averaged across components within a cluster. Right panel: Plot of 3D dipole source locations and their projections onto the MNI brain template. **(A)** lateral occipital (n = 16, s = 11), **(B)** right sensorimotor (n = 16, s = 16), **(C)** left sensorimotor (n = 16, s = 15), **(D)** medial parietal (n = 12, s = 12), **(E)** medial frontal (n = 11, s = 11), and **(F)** superior parietal (n = 10, s = 9) (representing the number of components n contributed by the number of subjects s within a cluster).

Figure 1 shows six neurophysiologically interpretable IC clusters observed relatively consistent across twenty-four subjects, including clusters located in or near the lateral occipital, right sensorimotor, left sensorimotor, medial parietal, medial frontal, and superior parietal regions. This figure shows the individual and mean scalp maps, the corresponding 3D dipole source locations, and their projections onto the MNI brain template for each cluster. The scalp maps of these components could often be modeled well by a single equivalent dipole. The mean residual variance or percentage difference of the IC projections from the theoretical projections of the model dipole across 81 components in six clusters was 3.67% (±3.03%). The largest cluster located in or near the lateral occipital area (Figure 1A) contained sixteen components from eleven subjects. Of these eleven subjects, seven contributed one component, three contributed two components from both the right and left sides, and one contributed three components. Two clusters located in or near the right and left sensorimotor cortices (Figures 1B and 1C) included sixteen components. Eleven subjects contributed to both the right and left sides, seven contributed to either the right or left side, and one contributed three components into the cluster. A cluster located in or near the medial parietal area (Figure 1D) contained twelve components from twelve subjects. A cluster located in or near the medial frontal area (Figure 1E) included eleven components from eleven subjects. The last cluster located in or near the superior parietal area (Figure 1F) contained ten components. Eight subjects contributed one component to the cluster, and one subject contributed two components. Table 1 details the number of components grouped into each cluster, the number of contributing subjects, and the Talairach coordinates of the centroid of the dipole distributions for each cluster. Furthermore, several studies [30,44-46] reported cortical ICs with equivalent dipole locations at or near frontal-central, right and left central, parietal and occipital lobes in various cognitive experiments.

**Table 1:** Major component clusters and the centroids of their source distributions

**Brain region**	**# Components**	**# Subjects (24)**	**Talairach coordinates**		
			**x**	**y**	**z**
Lateral occipital	16	11	34	-78	6
			-28	-79	6
Right sensorimotor	16	16	35	-26	45
Left sensorimotor	16	15	-29	-27	43
Medial parietal	12	12	2	-48	14
Medial frontal	11	11	1	12	35
Superior parietal	10	9	6	-56	64

**Figure 2:**
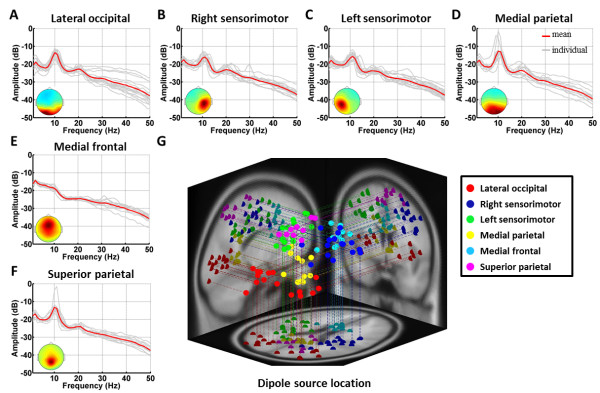
**Consistency of six independent component clusters.** The averaged and individual IC log-power spectra (dB) are plotted in red and gray lines, respectively, and the corresponding mean scalp maps of clusters are superimposed on the panels. **(A)** Lateral occipital cluster, **(B)** Right sensorimotor cluster, **(C)** Left sensorimotor cluster, **(D)** Medial parietal cluster, **(E)** Medial frontal cluster, and **(F)** Superior parietal cluster. **(G)** A 3D overview of equivalent dipole locations of the six clusters of interest and their projections onto the MNI brain template. Dots in the same color represent the components grouped into the same cluster.

Figure 2 shows the logarithmic spectral profiles of the six independent component clusters. Figure 2A-F plots the mean (red trace) and individual (gray traces) log spectral profiles of components for each of the six clusters. The figure shows that five of six component clusters exhibited a major peak in the alpha band (8-13 Hz) and a minor peak in the beta band (14-30 Hz), including lateral occipital (Figure 2A), right sensorimotor (Figure 2B), left sensorimotor (Figure 2C), medial parietal (Figure 2D), and superior parietal (Figure 2F) clusters. The medial frontal (Figure 2E) cluster exhibited a distinct spectral profile dominated by low-frequency activities. Figure 2G shows equivalent dipole locations for these clusters of interest in different colors. These results demonstrated that the resultant ICs within each cluster (shown in the same color) were highly comparable; each of them had a unique scalp map, dipole source location, and power spectrum.

### Musical structure-modulated brain activity

**Figure 3:**
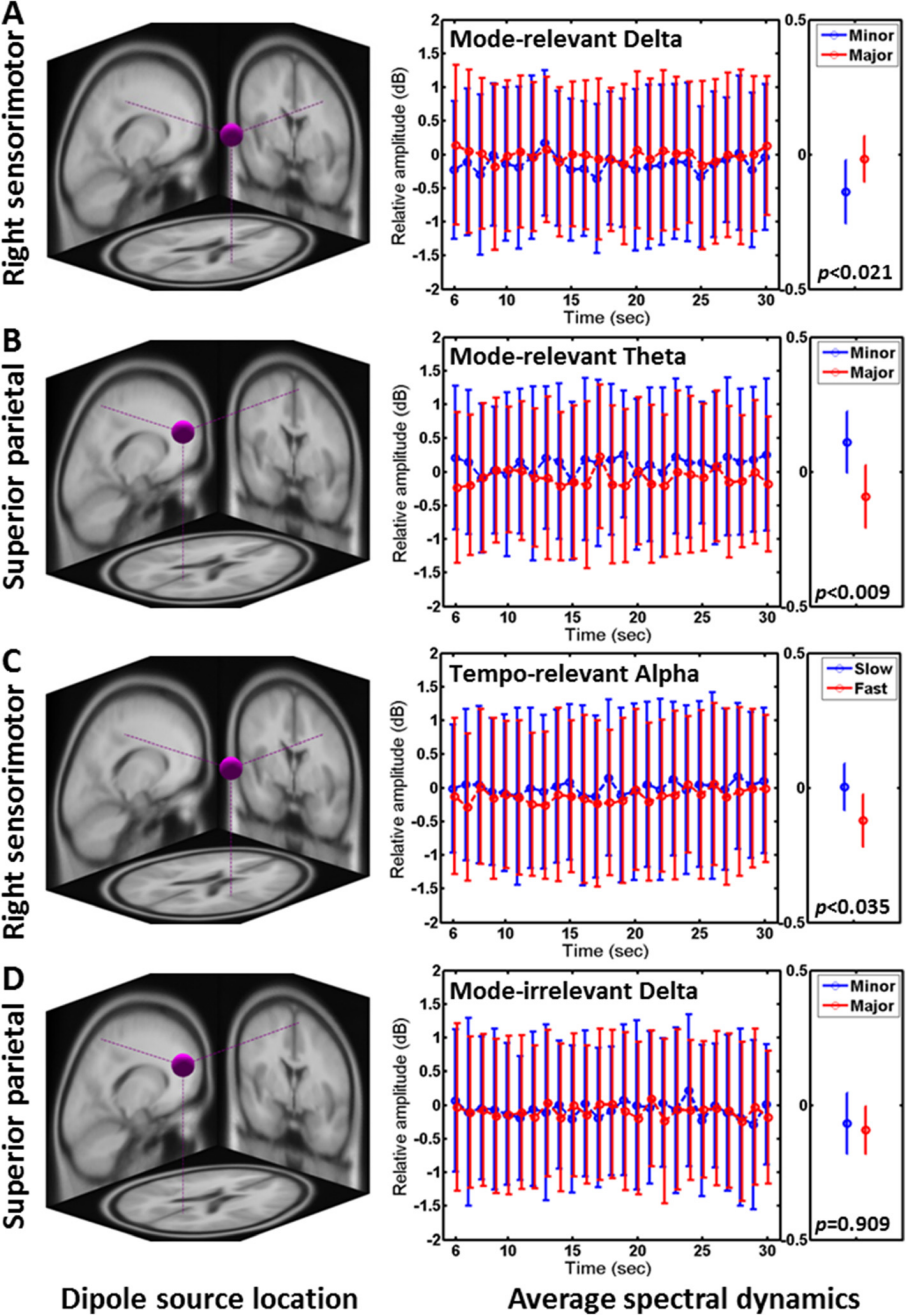
**Averaged spectral dynamics for IC clusters in response to musical mode and tempo.** (**A-D**, left) The centroids of equivalent dipole locations of selected IC clusters are projected onto the MNI brain template. (**A-D**, right) The averaged time courses (across music excerpts, left plot) and the averaged mean values of the time courses (across excerpts and time points, right plot) of cluster spectra. The red lines indicate the spectral changes in music with major mode/fast tempo, whereas the blue lines represent the spectral changes in music with minor mode/slow tempo. Note that the first five seconds of 30s music presentation was removed for spectral baseline correlation. Music in major mode significantly induced **(A)** higher right sensorimotor (x = 35, y = -26, z = 45) delta power (F_(1,12)_ = 7.15, *p* < 0.021) and **(B)** lower superior parietal (x = 6, y = -56, z = 64) theta power (F_(1,12)=_9.87, *p* < 0.009) versus music in minor mode. Music in fast tempo significantly induced **(C)** lower right sensorimotor alpha power versus music in slow tempo (F_(1,12)_ = 5.68, *p* < 0.035). **(D)** The superior parietal delta exhibited insignificance in response to musical mode changed (F_(1,12)=_0.01, *p* = 0.909), which was used for comparing the superior parietal theta **(B)**.

Figure 3 shows the spatio-spectral EEG dynamics associated with the changes in musical mode and tempo. In the right panel, the averaged spectral time courses (across music excerpts) and the averaged mean values of the spectral time courses (across excerpts and time points) of the IC clusters were used to demonstrate how component spectra differentially responded to distinct musical structures. Specifically, music in major mode augmented delta-band activity (F_(1,12)_ = 7.15, *p* < 0.021) over the right sensorimotor cortex (Figure 3A) and suppressed theta-band activity (F_(1,12)=_9.87, *p* < 0.009) over the superior parietal cortex (Figure 3B) as compared to minor mode. It might be worth noting that the medial frontal beta-band activity (not shown here) was also found to be marginally responsive to musical mode (F_(1,12)_ = 4.43, *p* = 0.057), resulting in a decreased beta-band power for major-mode music. Unlike musical mode, only the right sensorimotor alpha was found to be reactive to the musical tempo changes (F_(1,12)_ = 5.68, *p* < 0.035). That is, fast-tempo music excerpts induced low alpha-band activity, as compared to slow ones (Figure 3C). To further demonstrate the musical structure-modulated spectra, the superior parietal delta activity that exhibited insignificance in distinguishing musical mode (F_(1,12)=_0.01, *p* = 0.909) was shown at Figure 3D. As can be seen, unlike the superior parietal theta (Figure 3B), the time courses of delta-band power induced by minor- and major-mode music excerpts were highly merged along time. Note that the overlapping standard deviations of the spectral time courses between conditions were mainly attributed to the with- and between-subjects variability.

## Discussion

### Mode- and tempo-modulated spatio-spectral dynamics

Empirical results of this study showed that music with major mode tended to accompany the increased delta-band activity over the right sensorimotor cortex (*p* < 0.021, Figure 3A), the decreased theta activity over the superior parietal cortex (*p* < 0.009, Figure 3B), and the moderately decreased beta activity over the medial frontal cortex (*p* = 0.057) compared to minor-mode music, where fast-tempo music only engaged significant alpha suppression over the right sensorimotor cortex (*p* < 0.035, Figure 3C). Compared to the recent ICA-based music study [20], the present study not only fully explored the spatio-spectral dynamics across EEG frequency bands (delta, theta, alpha, beta, and gamma), but also took a step further to investigate how the spatio-spectral dynamics of different brain networks were modulated by the musical mode and tempo. The discussions below will compare the resultant EEG dynamics in terms of brain locations and the spectral dynamics in the literature. Due to the lack of EEG-based results in the literature, anatomical evidences previously provided by other neuroimaging modalities (such as fMRI, PET and MEG) are used to demonstrate the validity of resultant music-related brain sources. Table 2 lists brain areas of the present study that are either directly consistent with or supported by previous results.

**Table 2:** Summary of estimated dipole sources supported by previous music-related evidences

**Resultant dipole sources**	**Study**	**Modality**	**Brain regions**	**Music tasks**
Right sensorimotor	[10]	fMRI	Right middle frontal gyrus (BA 6)	Mode-Tempo
	[48]	fMRI	Right postcentral gyrus (BA 3/1/2)	Minor-Major
	[12]	MEG	Somatormotor cortex (BA 4/6)	Rhythm
Superior parietal	[48]	fMRI	Left inferior parietal lobule (BA 7)	Major-Minor
			Right inferior parietal lobule (BA 7)	Minor-Major
	[1]	PET	Right precuneus (BA 7)	Dissonance
	[7]	fMRI	Bilateral precuneus (BA 7)	Harmony
	[20]	EEG	Central area	Rhythm, Tonality
Medial frontal*	[11]	fMRI	Bilateral anterior cingulate gyrus (BA 24)	Minor-Major
	[10]	fMRI	Right anterior cingulate gyrus (BA 24)	Mode-Tempo

BA: Brodman area. *the resultant brain region of the present study marginally associated with musical structures (*p* = 0.057).

Regarding the musical mode-related activations, this study found the activations of the right sensorimotor delta, the superior parietal theta and the medial frontal beta were either significantly or moderately modulated by the processing of musical mode. As listed at Table 2, the previously reported findings in musical structure-related brain regions are able to validate the brain regions revealed by ICA. Firstly, the involvement of superior parietal region or precuneus has been reported to be associated with harmonic/consonant melodies [1,7], and may reflect the music-related mental state during music listening [47], such as memory retrieval or visual imagery. The degree of consonant harmonics are commonly considered simpler and more often in major keys, whereas dissonance is usually associated with minor mode [4,11]. Thus, the presence of the superior parietal region in the present study was not unexpected since all music excerpts used in this study, regardless of mode, were harmonious. In this study, the superior parietal region was found to significantly exhibit low theta-band activity as listening to music in major mode versus minor one (Figure 3B). Although a simultaneous PET and EEG study [47] reported the mode-related association in the beta band, the recent ICA-based music study [20] exactly revealed the same component spectra, namely a central cluster, distinctly engaged in the processing of musical mode, tonality and rhythm. Other music studies also reported the theta changes but tended to fluctuate over the frontal or central region [16,18].

Previous fMRI studies [10,11,48] supported the engagement of the medial frontal and the right sensorimotor activations in musical mode processing. The medial frontal activation during music listening has been suggested to reflect working memory of the perceptual context [12] and emotion perception [15,16]. This study found the medial frontal beta activity moderately decreased and the right sensorimotor delta activity increased as subjects listened to music in major mode (Figure 3A).

Regarding the EEG correlates of musical tempo, this study only found the activation over the right sensorimotor cortex significantly responded to distinct musical tempi, consistent with previous fMRI and MEG studies [10,12,48]. Music has been suggested to engage a physical entrainment of motor and physiological function [49]. This effect could be mediated through sensory–motor feedback circuits. For example, fast and high-pitched music is typically perceived as lively and is associated with rapid and high-energy movement whereas slow and low-pitched music has a calming effect that induces sadness and is associated with slow and low-intensity movements [8,49]. The perception of musical rhythm has also been suggested to activate the somatomotor cortex without physical movements [12]. Our study showed that fast-tempo music excerpts induced a significant suppression in the alpha power over the right sensorimotor cortex, as compared to slow ones (Figure 3C). The alpha activity projected from the sensorimotor region was generally referred to as the mu rhythm, which has a well-established role in motor imagery [50] and engaged mirror neuron system during music listening [51]. The alpha suppression further reflects the engagement of sensory input or motor preparation/production [52]. Accordingly, the fast-tempo-induced sensorimotor alpha suppression of the present study might imply that the musical tempo (beats per minute) drive motor imagery to some extent. Such musical tempo-driven sensorimotor alpha suppression has not been reported in the recent ICA-based music study [20]. In addition, an early EEG study [13] reported that changes in either tempo or mode would result in a frontal alpha-power asymmetry (F3 versus F4). Neither the current study nor the recent ICA-based study [20] found the involvement of the frontal alpha activities in music processing. One possible explanation is that the spectral dynamics revealed by ICA substantially differed from the conventional EEG analysis.

Although some deep brain structures included the limbic and paralimbic areas are reportedly responsive to music perception [1,7,11], the current result did not support this association. A possible explanation is the limited accessibility of EEG to deep brain structures. Unlike fMRI and PET, EEG recorded from scalp sensors is less sensitive to the activities arising from the deep brain structures.

Music is considered an ecologically valid auditory stimulus and would lead to EEG patterns evolving over time during music listening [16,53]. It is reasonable to expect that two distinct temporal waveforms could be observed from an individual while listening to two different, for example, fast-tempo music. Once involving more music excerpts and individuals for the analysis, the within- and between-subjects variability in music-listening task very likely introduced such variability in spectral time courses of interest (*c.f.* Figure 3).

### Validity and significance of independent component analysis

The rationale of exploring neural correlates of music appreciation using activities of independent components as opposed to those of scalp channels is that due to volume conduction through cerebrospinal fluid, skull, and scalp, EEG signals collected from the scalp are supervisions (or mixtures) of neural and artifactual activities from multiple brain or extra-brain processes occurring within a large volume. The decomposed independent components exhibit the activity arising from a specific brain or extra-brain network and allow us to explore the neural dynamics of the source induced by different musical features without being confounded by other irrelevant source activities. Several studies [27-29] have demonstrated the effectiveness of ICA in improving SNR of activities of interest. Furthermore, as music research moved from well-controlled laboratory setting to real-life applications, EEG collected from the scalp electrodes will unavoidably comprise variety of signal sources arising from music-relevant activities, music-irrelevant activities, and movement and environment artifacts. The artifactual and music-irrelevant signals might even fluctuate within the same frequency range that used for characterizing music-modulated dynamics of interest, making the analysis very difficult, if ever possible. Therefore, it would be crucial to develop spatial filters that can separate the spatio-spectral features of sources of interest from other irrelevant brain signals and artifacts to assess music-related EEG dynamics.

With regard to the inter-subject reproducibility of ICs as reported in [29], in general the IC clusters accounting for phase-, stimulus-, or response-locked event-related potential would be largely replicated in many subjects, whereas ICs accounting for non-phase locked EEG activities varied across subjects. For example, a recent study, which utilized ICA to investigate motion-sickness-related brain responses (non-phase locked EEG activities) across 19 subjects during a simulated driving task [30], reported that the inter-subject reproducibility of five component clusters ranged from 58% to 63% (percentage of contributing subjects). Music perception is rather subjective and might involve very complex brain networks in response to perception and integration of different aspects of music. Therefore, the inter-subject reproducibility of 38-67% across six clusters in the current study seems acceptable. Furthermore, it is noted that differences in the cortical anatomy might also contribute to inter-subject variability. For example, the two spatially fixed brain sources with different projection directions can generate distinct EEG distributions over the scalp. This issue not only exhibited in the source-based analysis, *e.g.*, ICA, but also presented in channel-based analysis [46]. However, it is worth noting that the EEG dynamics associated with musical mode or tempo only apply to the subjects’ components contributing to the clusters (see Figure 1). For example, 16 of 24 subjects (67%) exhibited music-related brain dynamics in the right sensorimotor cluster, whereas only 9 subjects (38%) exhibited music-related brain dynamics in the superior parietal cluster. Therefore, interpretations of the involvements of these component clusters need to take the individual difference into account. In the future study, the use of a high-density EEG cap might in part alleviate the reproducibility issue caused by cognitive and structural differences. It has been suggested that using more channels, *e.g*., 256, for ICA decomposition would produce more neurophysiologically feasible components [23].

Although the present study successfully applied ICA to decompose the EEG segments recorded during the music listening task and found consistent independent brain processes across multiple subjects, unfortunately, the estimation of dipole location based on individual component activation map was far from precise and caused noticeable deviations in the source localization results across different subjects. The reasons are listed as follows. First, since the dipole localization results were only based on 32 scalp channels, estimation error was inevitable in such a low-density montage [54]. Second, due to lack of precise 3D sensor locations and anatomical images from each individual, using standard sensor locations and anatomical templates probably increased errors in source locations. Therefore, Table 1 only gives the approximate source locations of the involved cortical source patches.

### Limitation

Evidence indicates that musical mode and tempo are the two major musical structures that affect perceived emotional valence [5,9,10]. Therefore, the current study explored the more fundamental question of how objective musical structures (*i.e.*, tempo and mode) correspond with brain responses when listening to a movie soundtrack. However, a caveat of this study is that the movie soundtracks presented to the subjects were not specifically composed with well controlled mode and tempo for manipulating the brain responses with specific musical structures. That is, the music samples did not contain fixed mode with only variant tempo or vice versa. Variable musical parameters may have caused confounding effects in the EEG data. Nevertheless, this trade-off between experimental and real-life environments is acceptable because using highly controlled music excerpts to study the link between musical structures and the corresponding human brain responses may lack the ecological validity that unfolds when actually listening to music [11,17]. Nonetheless, although the music excerpts were not highly controlled in our study, the spatio-spectral brain processes that exhibited statistically significant changes as the subjects listened to music in different mode or tempi were consistent with the neuroimaging literature. Thus, the result of current study may shed light on the new exploration of music-listening responses using scalp EEG.

## Conclusions

By applying ICA to multi-channel scalp EEG data, this study explored temporally independent brain sources that contribute to the perception of musical mode and tempo during natural music listening. The results of this study showed that changes in musical mode and tempo involve several brain areas identified in previous studies using other neuroimaging modalities. More importantly, this study systematically explored how changes in musical mode and tempo affect the EEG spectral characteristics of different brain networks. The study, in conjunction with advances in newly available dry and wireless EEG technology, might lead to real-life applications for music therapy, training and entertainment in naturalistic environments.

## Abbreviations

ANCOVA: The analysis of covariance; BEM: Boundary element head model; BOLD: Blood-oxygen-level dependent; EEG: Electroencephalography; EOG: Electrooculogram; fMRI: Functional magnetic resonance imaging; IC: Independent component; ICA: Independent component analysis; MEG: Magnetoencephalography; MNI: Montreal neurological institute; PET: Positron emission tomography; rCBF: Regional cerebral blood flow; STFT: Short-time Fourier transform; SNR: Signal-to-noise ratio.

## Competing interests

The authors declare that they have no competing interests.

## Authors’ contributions

YPL undertook the data collection, analyzed the data, interpreted the results and drafted the manuscript. JRD helped to interpret the results and revise the manuscript. WF helped to conceive the statistical analysis and interpret the statistical results. JHC contributed to the experimental design and provided critical comments. TPJ helped to interpret the results, revise the manuscript and provide critical comments. All authors read and approved the final manuscript.
